# The Changing Landscape of Hantavirus Infections: A Narrative Review of Epidemiology, Pathogenesis, and Countermeasures

**DOI:** 10.1002/rmv.70191

**Published:** 2026-07-31

**Authors:** Francesco De Maria, Francesco Branda, Giancarlo Ceccarelli, Fabio Scarpa, Massimo Ciccozzi, Alessandro Russo

**Affiliations:** ^1^ Infectious and Tropical Diseases Unit Department of Medical and Surgical Sciences ‘Magna Graecia’ University of Catanzaro Catanzaro Italy; ^2^ Unit of Medical Statistics and Molecular Epidemiology Università Campus Bio‐Medico di Roma Rome Italy; ^3^ Department of Public Health and Infectious Diseases University Hospital Policlinico Umberto I Sapienza University of Rome Rome Italy; ^4^ Department of Biomedical Sciences University of Sassari Sassari Italy

**Keywords:** andes virus, climate change, endothelial dysfunction, haemorrhagic fever with renal syndrome, hantavirus cardiopulmonary syndrome, hantaviruses, monoclonal antibodies, one health, person‐to‐person transmission, zoonotic spillover

## Abstract

Hantaviruses are emerging zoonotic pathogens of increasing global public health significance. Their epidemiological landscape is rapidly changing due to ecological disruption, climate variability, urbanisation, and evolving human–animal interfaces. This narrative review synthesises current knowledge on hantavirus virology, epidemiology, pathogenesis, clinical management, and emerging countermeasures, with a focus on developments from the past 5 years. Traditionally classified into Old World viruses causing haemorrhagic fever with renal syndrome (HFRS) and New World viruses causing hantavirus cardiopulmonary syndrome (HCPS), this dichotomy has become increasingly blurred, with overlapping renal and pulmonary manifestations now recognized. Among the most consequential recent advances is the consolidated evidence for person‐to‐person transmission of Andes virus, documented among household contacts and during the prodromal phase, with viral shedding in respiratory secretions. Endothelial dysfunction remains the pathological hallmark of severe disease, driven by VEGF sensitisation, Src kinase and RhoA pathway activation, pericyte infection, and dysregulated inflammatory responses including IL‐6 *trans*‐signalling. Concurrently, environmental and ecological studies have linked climate change, rodent population dynamics, land‐use modification, and urbanisation to increased human disease risk across multiple continents. Diagnostic strategies continue to rely on serology and molecular testing, while management remains predominantly supportive, with extracorporeal membrane oxygenation (ECMO) improving survival in fulminant HCPS. Importantly, recent structural virology advances have enabled the development of broadly neutralising monoclonal antibodies targeting conserved quaternary epitopes, as well as next‐generation vaccine platforms including DNA, mRNA, and prefusion‐stabilised glycoprotein candidates. Hantaviruses represent a paradigmatic One Health challenge at the intersection of environmental change, viral evolution, and global interconnectedness. Despite major progress in understanding transmission dynamics and endothelial pathogenesis, critical gaps remain regarding determinants of person‐to‐person spread, long‐term post‐infection sequelae, and the absence of licenced broadly protective vaccines or specific antiviral therapies. Future preparedness will require integrated surveillance frameworks combining ecological monitoring, genomic epidemiology, and clinical readiness within coordinated international public health strategies.

**Abbreviations**
AKI: acute kidney injury
ANDV: Andes virus
CDC: Centres for Disease Control and Prevention
DAF: decay‐accelerating factor
DOBV: Dobrava‐Belgrade virus
ECMO: extracorporeal membrane oxygenation
Gc: glycoprotein Gc
Gn: glycoprotein Gn
HCPS: hantavirus cardiopulmonary syndrome
HFRS: haemorrhagic fever with renal syndrome
HTNV: Hantaan virus
IFN: interferon
IFN‐γ: interferon‐gamma
IL‐6: interleukin‐6
IL‐15: interleukin‐15
IL‐6R: interleukin‐6 receptor
IRF: interferon regulatory factor
M: medium (RNA segment)
mRNA: messenger ribonucleic acid
N: nucleocapsid protein
NKG2D: natural killer group 2 member D
PAI‐1: plasminogen activator inhibitor‐1
PPE: personal protective equipment
PUUV: Puumala virus
RdRp: RNA‐dependent RNA polymerase
RIG‐I: retinoic acid‐inducible gene I
RT‐PCR: reverse transcription polymerase chain reaction
S: small (RNA segment)
SEOV: Seoul virus
sIL‐6R: soluble interleukin‐6 receptor
SNV: Sin Nombre virus
sTM: soluble thrombomodulin
TLR4: Toll‐like receptor 4
TNF‐α: tumour necrosis factor alpha
TRAF6: TNF receptor‐associated factor 6
VEGF: vascular endothelial growth factor
WHO: World Health Organization

## 1 Introduction

Hantaviruses are negative‐sense RNA viruses belonging to the family *Hantaviridae* (order *Bunyavirales*) that are naturally maintained in persistent infections within rodent, insectivore, and bat reservoirs. As rodent‐borne zoonotic pathogens, they continue to pose an important and evolving global public health threat, causing two major clinical syndromes: haemorrhagic fever with renal syndrome (HFRS) in Eurasia and hantavirus cardiopulmonary syndrome (HCPS) in the Americas. Although historically considered geographically restricted infections associated with predictable seasonal and occupational risk patterns, the epidemiological landscape of hantavirus disease is changing rapidly. Increasing ecological disruption, urban expansion, climate variability, globalisation of trade and travel, and improved molecular surveillance have all contributed to the recognition of hantaviruses as dynamic emerging pathogens with substantial epidemic potential [1, 2, 3, 4, 5].

Old World hantaviruses, including Hantaan (HTNV), Dobrava‐Belgrade (DOBV), Seoul (SEOV), and Puumala (PUUV) viruses, are primarily associated with renal manifestations ranging from mild nephropathia epidemica to severe HFRS with haemorrhagic complications, thrombocytopaenia, and acute kidney injury. By contrast, New World hantaviruses such as Andes (ANDV) and Sin Nombre (SNV) viruses predominantly cause HCPS, a rapidly progressive disease characterised by capillary leak, non‐cardiogenic pulmonary oedema, circulatory collapse, and high mortality rates ranging from 30% to 40% [1, 6, 7, 8]. However, this traditional clinical dichotomy has become increasingly blurred over the past decade. Renal abnormalities, including proteinuria and acute kidney injury, are frequently observed in HCPS and have been independently associated with mortality, whereas pulmonary involvement can occur in severe Old World infections, particularly those caused by HTNV and DOBV. These observations collectively support the concept that hantavirus disease represents a spectrum of endothelial dysfunction rather than two completely distinct clinical entities [1, 6, 9, 10].

Among the most consequential developments of the last decade has been the consolidation of evidence supporting person‐to‐person transmission of Andes virus, a feature that distinguishes ANDV from all other known hantaviruses. Unlike classical rodent‐borne exposure events, Andes virus can spread between humans, particularly among close household contacts, intimate partners, and caregivers during the prodromal phase of infection, often before the onset of severe cardiopulmonary manifestations [11, 12, 13]. Prospective virological studies have demonstrated prolonged viral shedding in respiratory secretions and saliva, while experimental models using Syrian hamsters have confirmed transmissibility under controlled conditions [13, 14, 15]. These observations have fundamentally transformed the conceptual framework of hantavirus epidemiology, raising concerns regarding outbreak preparedness, infection prevention in healthcare settings, and the potential for healthcare‐associated transmission. The recent World Health Organization report describing a multinational hantavirus cluster linked to cruise ship travel further illustrates the continued relevance of robust surveillance systems and rapid international coordination [16].

Concurrently, advances in structural virology, immunology, and translational research have significantly improved understanding of hantavirus pathogenesis at the molecular and cellular levels. Endothelial permeability remains the central pathological hallmark of severe disease, but recent work has highlighted increasingly complex interactions involving innate immune activation, T‐cell responses, cytokine dysregulation, pericyte infection, vascular signalling pathways, and viral immune evasion mechanisms [17, 18, 19, 20, 21, 22, 23, 24]. The discovery that hantaviruses infect pericytes, cells that stabilise microvessel walls, has provided new insights into the mechanisms underlying capillary leak. Furthermore, pathogenic hantaviruses have been shown to sensitise endothelial cells to vascular endothelial growth factor (VEGF), activate Src family kinases and RhoA‐dependent cytoskeletal rearrangements, and dysregulate IL‐6 *trans*‐signalling. These mechanistic insights are now driving the development of novel therapeutic strategies, including broadly neutralising monoclonal antibodies targeting conserved quaternary epitopes, host‐directed therapies aimed at stabilising endothelial barrier function, and next‐generation nucleic acid vaccine platforms [25, 26, 27, 28, 29, 30, 31, 32, 33, 34].

At the same time, environmental and ecological studies increasingly support the role of climate change, biodiversity shifts, land‐use modification, habitat fragmentation, and urbanisation in shaping hantavirus transmission dynamics at local, regional, and continental scales. Rodent population fluctuations associated with temperature anomalies, precipitation patterns, mast seeding events, and landscape transformation have been linked to increased human disease risk across Europe, Asia, and the Americas [2, 35, 36, 37, 38, 39, 40, 41, 42]. The emergence of Seoul virus in urban rat populations worldwide, including detection in seaports, slums, and metropolitan areas, and the identification of imported or pet rats as sources of human infection emphasise the growing importance of urban zoonotic interfaces. These findings challenge the traditional view of hantaviruses as exclusively rural pathogens and underscore the need for surveillance systems that encompass both sylvatic and synanthropic reservoir populations [43, 44, 45].

In Europe, hantavirus disease remains substantially underrecognized despite accumulating evidence of widespread circulation. Puumala virus continues to account for the majority of diagnosed infections, particularly in Northern and Central Europe, where nephropathia epidemica represents one of the most common zoonotic viral infections, Notably, the ecological drivers of bank vole population cycles differ between these regions: in Northern Europe (Finland, Sweden, Norway), cycles are chiefly predator‐driven and recur roughly every 3–4 years, whereas in Central Europe outbreaks are more closely tied to mast seeding of oak and beech, with intervals that have varied historically but have more recently occurred approximately every 2–3 years. Nevertheless, increasing recognition of Seoul virus circulation in urban rat populations across multiple European countries, expanding ecological suitability linked to climate change, and accumulating evidence suggesting substantial underdiagnosis in Southern Europe, including Italy, Spain, and Greece, indicate that the true burden of hantavirus infection in Europe is likely substantially underestimated. Serological and ecological evidence from Italy suggests that autochthonous circulation of orthohantaviruses is broader than generally appreciated, although confirmed cases remain infrequently reported due to limited clinical awareness and diagnostic testing [8, 37, 41, 43, 46, 47].

This Review summarises recent advances in hantavirus epidemiology, ecology, transmission dynamics, clinical manifestations, immunopathogenesis, diagnostics, management, and emerging countermeasures, with particular emphasis on developments from the past 5 years (2021–2026). We also discuss unresolved questions regarding the molecular determinants of endothelial injury, predictive biomarkers for severe disease, the basis of person‐to‐person transmission of Andes virus, and preparedness strategies in the context of accelerating environmental change. Section 2 presents hantavirus virology and structural biology, covering genome organization, glycoprotein architecture, mechanisms of cell entry, and viral replication. Section 3 examines environmental drivers, ecological dynamics, and One Health dimensions, including reservoir dynamics, climate‐sensitive transmission, urbanisation, and biodiversity. Section 4 analyzes outbreak investigation, contact tracing, and person‐to‐person transmission of Andes virus, including household clusters, serial interval analysis, and healthcare‐associated exposure assessment. Section 5 integrates the clinical, pathogenetic, diagnostic, and therapeutic dimensions of hantavirus disease, covering the clinical spectrum of HFRS and HCPS, immunopathogenesis and endothelial dysfunction, diagnostic approaches and surveillance systems, critical care management including ECMO, and emerging countermeasures such as monoclonal antibodies and next‐generation vaccine platforms. Finally, Section 6 concludes by highlighting persistent knowledge gaps and future research priorities, emphasising the need for integrated One Health approaches that bridge wildlife ecology, climate science, molecular epidemiology, and clinical preparedness.

## 2 Virology and Structural Biology: From Genome Organization to Endothelial Dysfunction

Hantaviruses are enveloped, negative‐sense RNA viruses belonging to the order *Bunyavirales* and the family *Hantaviridae* (subfamily Mammantavirinae, genus *Orthohantavirus*). Their genome is tripartite, consisting of three single‐stranded RNA segments designated small (S), medium (M), and large (L). The S segment encodes the nucleocapsid (N) protein, which encapsidates the viral RNA and plays critical roles in viral replication, assembly, and modulation of host innate immune responses. The *M* segment encodes a single glycoprotein precursor that is post‐translationally cleaved into the two envelope glycoproteins Gn and Gc, which form heterodimers and mediate host cell attachment and membrane fusion. The *L* segment encodes the RNA‐dependent RNA polymerase (RdRp), which is responsible for viral transcription and genome replication [5, 17]. Viral diversity is extensive and reflects long‐standing co‐evolutionary relationships with rodent, insectivore, and bat hosts, although rodents remain the principal reservoirs associated with human disease. This co‐evolution has resulted in a remarkable degree of host specificity, with most hantavirus species tightly adapted to a single reservoir species [3, 48, 49].

Ordered, interlaced lattices on the virion surface, with Gn and Gc heterodimers assembling into tetrameric spikes. During cell entry, these spikes undergo substantial conformational rearrangements that expose the fusion loops of Gc and facilitate membrane merger [17, 18]. Earlier biochemical and antibody‐competition studies had already implicated multiple steps of the entry pathway, that is, receptor engagement, conformational transitions, and membrane fusion, as targets of neutralising antibodies; more recent cryo‐electron microscopy and antibody epitope mapping have confirmed and extended these findings to near‐atomic resolution, helping to visualise rather than newly discover the mechanistic basis of neutralisation. Importantly, these structural studies have identified conserved quaternary epitopes, structural determinants formed by the juxtaposition of Gn and Gc, that are shared across multiple hantavirus species. These conserved epitopes may support the development of pan‐hantavirus immunotherapies and broadly protective vaccines [28, 31, 50].

Hantavirus entry primarily targets endothelial cells, which line the inner surface of blood vessels and regulate vascular permeability. However, additional cell types may also contribute to disease pathogenesis, including epithelial cells (particularly in the renal tubules and pulmonary epithelium), macrophages and dendritic cells (which may serve as vehicles for viral dissemination), and pericytes, contractile cells that surround endothelial cells and stabilise microvessel walls. Infection of pericytes by Andes virus has been shown to directly enhance endothelial cell permeability, providing a novel mechanism for capillary leak independent of direct endothelial infection [23, 51, 52, 53].

A diverse array of cellular receptors and attachment factors have been proposed for hantavirus entry, although the identity of the bona fide receptor(s) remains unresolved. Integrins, particularly β3 integrins for pathogenic hantaviruses and β1 integrins for non‐pathogenic strains, were long proposed as entry receptors; however, this model remains controversial, as several laboratories have been unable to reproduce the original findings, and a role for β2 integrins has also been suggested. Decay‐accelerating factor (DAF/CD55) and protocadherin‐1 have more recently emerged as promising receptor or attachment‐factor candidates, and additional surface molecules have also been implicated as potential co‐receptors. Importantly, receptor dependence appears to differ between Old World and New World hantaviruses, which may explain differences in tissue tropism and clinical presentation. Experimental depletion studies using CRISPR‐Cas9 and RNA interference approaches have further suggested that highly pathogenic hantaviruses exploit distinct endothelial entry pathways compared with non‐pathogenic strains, potentially contributing to differences in virulence and the severity of vascular injury [32, 54].

Following receptor‐mediated endocytosis and pH‐dependent fusion in late endosomes, the viral ribonucleoprotein complex is released into the cytoplasm, where replication and transcription occur exclusively in the cytoplasmic compartment. The viral RdRp initiates transcription via a cap‐snatching mechanism, stealing short 5′ capped RNA fragments from host messenger RNAs to prime viral mRNA synthesis. This process is closely intertwined with host innate immune signalling pathways, as the depletion of host mRNAs may simultaneously dampen antiviral responses. Hantaviruses have been proposed to modulate interferon (IFN) responses and antiviral signalling through several candidate mechanisms, including inhibition of IFN regulatory factors (IRFs), interference with signalling adaptor molecules such as MAVS and STING, and degradation of host antiviral proteins; however, the evidence supporting these mechanisms is derived largely from in vitro overexpression systems, and the extent to which they operate during natural infection remains to be firmly established [20, 21, 22]. Recent comparative studies have demonstrated marked species‐specific differences in innate immune activation between reservoir hosts (rodents) and incidental hosts (humans). Rodent reservoir cells mount a tightly regulated and transient IFN response that limits viral replication without causing immunopathology, allowing persistent infection. In contrast, human cells often exhibit either delayed or exaggerated IFN responses, contributing to viral dissemination and immune‐mediated tissue injury. These differences help explain the ability of rodents to sustain persistent, asymptomatic infection without developing overt disease [24, 52, 53, 55].

The molecular determinants of hantavirus virulence remain incompletely understood, representing an active area of investigation. Unlike many other haemorrhagic fever viruses that cause widespread cytopathic cell death, pathogenic hantaviruses do not induce substantial endothelial cell lysis. Instead, increasing evidence indicates that they directly modulate endothelial permeability pathways through subtle alterations of cell signalling rather than through overt cell death. VEGF‐mediated signalling has been shown to be potentiated in hantavirus‐infected endothelial cells, leading to increased vascular permeability. RhoA, a small GTPase that regulates cytoskeletal dynamics, is activated following hantavirus infection and promotes the formation of stress fibres and intercellular gaps. Src family kinases, which phosphorylate components of endothelial adherens junctions (such as vascular endothelial‐cadherin), are also activated and contribute to junctional disassembly. The convergence of these signalling pathways results in dysregulation of endothelial barrier integrity and increased capillary leak [23, 56, 57, 58, 59, 60, 61]. Collectively, these findings reinforce the concept that severe hantavirus disease is fundamentally a disorder of vascular dysfunction and immune‐mediated capillary leak, rather than a primary cytolytic viral infection. This insight has profound implications for the development of targeted therapies, shifting the focus from direct antiviral agents to host‐directed interventions that stabilise endothelial barrier function and modulate inflammatory responses.

## 3 Environmental Drivers, Ecological Dynamics, and the One Health Continuum

The epidemiology of hantavirus infection is shaped by complex, multi‐scale interactions between reservoir host population dynamics, environmental conditions, human behaviour, land‐use patterns, and socio‐economic determinants. Unlike directly transmitted pathogens, hantaviruses do not require humans to complete their life cycle; they are maintained indefinitely within rodent, insectivore, and bat reservoirs, and human infection is an incidental spillover event rather than a necessary step in viral persistence. The probability of such spillover depends on the density and prevalence of infection in reservoir populations, the degree of rodent‐human interface, and environmental factors that influence both. Increasingly, hantavirus disease is being recognized as a paradigmatic One Health challenge in which climatic, ecological, and anthropogenic drivers converge to influence spillover risk. This integrated perspective acknowledges that human health cannot be separated from the health of wildlife populations and the ecosystems they inhabit [2, 35, 36, 37, 48, 62].

Globally, the burden of hantavirus disease remains difficult to quantify because of substantial underdiagnosis, limited surveillance systems in many endemic regions, marked geographic heterogeneity in diagnostic capacity, and the non‐specific nature of early symptoms, which are often mistaken for influenza, dengue, leptospirosis, or other febrile illnesses [4, 25]. A summary of the major hantaviruses associated with human disease, including their reservoir hosts, geographic distribution, clinical syndromes, and transmission characteristics, is provided in Table 1, whereas Table S1 reports confirmed case numbers by region, country, and virus over the past decade. Nevertheless, endemic regions continue to report recurrent outbreaks and expanding ecological niches. In the Americas, Andes virus (ANDV) and Sin Nombre virus (SNV) remain the principal causes of hantavirus cardiopulmonary syndrome (HCPS), while Hantaan (HTNV), Puumala (PUUV), Dobrava‐Belgrade (DOBV), and Seoul (SEOV) viruses account for the majority of haemorrhagic fever with renal syndrome (HFRS) cases across Eurasia. Notably, SEOV has a global distribution due to the worldwide spread of its reservoir hosts, *Rattus norvegicus* and *Rattus rattus* [1, 6, 7, 8, 63].

**TABLE 1:** Major hantaviruses associated with human disease.

Virus	Main reservoir host	Geographic distribution	Main clinical syndrome	Human‐to‐human transmission	Reported case fatality rate
Andes virus (ANDV)	*Oligoryzomys longicaudatus*	Chile, Argentina	Hantavirus cardiopulmonary syndrome (HCPS)	Confirmed	Up to 35%–40%
Sin nombre virus (SNV)	*Peromyscus maniculatus*	North America	HCPS	Not confirmed	Approximately 30%–40%
Hantaan virus (HTNV)	*Apodemus agrarius*	China, Korea, far East Russia	Haemorrhagic fever with renal syndrome (HFRS)	Not confirmed	Approximately 5%–15%
Seoul virus (SEOV)	*Rattus norvegicus*, *Rattus rattus*	Worldwide urban distribution	HFRS	Not confirmed	Usually < 2%
Puumala virus (PUUV)	*Clethrionomys glareolus*	Northern and Central Europe	Nephropathia epidemica	Not confirmed	< 1%
Dobrava‐belgrade virus (DOBV)	*Apodemus flavicollis*	Balkans and Eastern Europe	Severe HFRS	Not confirmed	Up to 10%–12%
Laguna negra virus	*Calomys laucha*	Paraguay, Bolivia	HCPS	Suspected but unconfirmed	Variable
Rio Mamoré virus	Rodent reservoirs under investigation	Amazon basin	HCPS‐like disease	Not confirmed	Unknown

*Note:* Data on virus–reservoir associations, geographic distribution, and clinical syndromes are derived from epidemiological surveillance studies, outbreak investigations, and recent reviews [1, 5, 8, 48, 49, 63, 64, 65]. Human‐to‐human transmission is confirmed only for Andes virus [11], while Laguna Negra virus has suspected but unconfirmed transmission. Reported case fatality rates may vary according to geographic setting, healthcare access, diagnostic capacity, and outbreak characteristics [5, 8, 43, 47, 65]. Seoul virus has been increasingly documented in worldwide urban settings, including emerging recognition in Europe [43, 47].

South America represents one of the most dynamic regions for hantavirus emergence, with multiple pathogenic species described over the past 3 decades. Long‐term surveillance data from Panama have highlighted the persistence of endemic transmission over more than 2 decades, with changing patterns of rodent ecology and human exposure. These long‐term datasets have revealed that outbreaks are often preceded by environmental conditions favouring rodent population booms, suggesting the potential for predictive modelling [63]. Argentina and Chile continue to experience periodic Andes virus outbreaks, particularly in rural and forested regions of the Andean foothills and Patagonia, where environmental conditions, including rainfall patterns, temperature, and vegetation cover, favour reservoir amplification. Person‐to‐person transmission of ANDV has added a layer of complexity to outbreak response in these regions [12, 13]. Recent work from Peru additionally suggests previously underrecognized endemic circulation in Amazonian ecosystems, emphasising the likelihood that hantavirus diversity and geographic distribution remain incompletely mapped in Latin America. This is particularly concerning given the region's high biodiversity and ongoing deforestation, which may increase rodent‐human interfaces [52].

In North America, Sin Nombre virus remains the dominant cause of HCPS, particularly in the western United States and Canada, where its primary reservoir, the deer mouse (*Peromyscus maniculatus*), is abundant. Ecological studies increasingly link disease incidence to arid climates, open developed landscapes, and rodent population fluctuations associated with climatic variability, particularly precipitation patterns that influence vegetation growth and food availability for rodents. Warmer, drier conditions followed by periods of adequate rainfall have been associated with deer mouse population irruptions and subsequent human cases [39, 66]. Climate‐driven amplification of reservoir populations may provide opportunities for predictive early warning systems, as suggested by surveillance studies from endemic regions of Argentina, where rapid synchronous increases in rodent abundance have been shown to anticipate HCPS outbreaks by several weeks to months [38].

In Europe, Puumala virus is the most prevalent hantavirus infecting humans and is responsible for large seasonal outbreaks of nephropathia epidemica, especially in Northern and Central Europe (Finland, Sweden, Germany, France, Belgium, and the Netherlands). The bank vole (*Clethrionomys glareolus*), the reservoir of PUUV, exhibits multiannual population cycles whose drivers differ by latitude. In Northern Europe (Finland, Sweden, Norway), cycles of roughly 3–4 years are thought to be predominantly predator‐driven (top‐down regulation), whereas in Central Europe (e.g., Germany, France, Belgium) outbreaks track mast seeding of oak and beech, with historical intervals of several years that have more recently shortened to approximately every 2–3 years. These population dynamics are consistently followed by increased human disease incidence, and Finland, where PUUV was first identified, reports the highest incidence of human hantavirus infection in Europe [8, 9, 65]. However, recent ecological and epidemiological analyses suggest that the European landscape of hantavirus infection is evolving rapidly. Climate change is altering the distribution and phaenology of both reservoir hosts and their habitats; biodiversity shifts may disrupt predator‐prey relationships that normally regulate rodent populations; altered forest ecology (including changes in tree species composition and forest management practices) affects food availability; and changes in rodent community composition influence viral circulation dynamics [37, 39, 40, 41, 42].

Several studies have demonstrated robust associations between temperature anomalies, precipitation patterns, mast seeding events, and increased incidence of human infection across European countries. Warmer winters, in particular, have been associated with higher overwinter survival of bank voles, leading to larger spring populations and earlier, more intense transmission seasons. A pan‐European assessment that integrated climate data, biodiversity metrics, socio‐economic factors, and human disease reports identified climate variables (temperature and precipitation), biodiversity (rodent species richness and predator abundance), and socio‐economic factors (agricultural land use, forest cover, and population density) as major determinants of human risk. These findings support the need for integrated surveillance systems that combine environmental and public health data, moving beyond simple case reporting to incorporate ecological indicators [35, 37, 39, 40, 41]. Likewise, ecological studies from Western Europe suggest that environmental determinants, including landscape fragmentation, proximity to forests, and land‐use intensity, may strongly influence the spatial distribution of nephropathia epidemica, creating localized hotspots of transmission even within low‐incidence countries [41].

Urbanization has emerged as another important dimension of hantavirus epidemiology, challenging the traditional view of hantaviruses as exclusively rural pathogens. Seoul virus, historically associated with *Rattus norvegicus* and *Rattus rattus*, has increasingly been detected in urban environments worldwide, including seaports, inner‐city slums, public housing complexes, and subway systems. The global movement of goods via shipping routes has facilitated the spread of infected rats across continents, establishing SEOV as a truly global pathogen [43, 44, 45]. Human infections linked to pet rats have now been documented in several countries, including the United States, Canada, the United Kingdom, and Germany, highlighting the growing importance of non‐traditional transmission settings. Pet rat‐associated outbreaks have been traced back to commercial breeding facilities, where infected rats are distributed across wide geographic areas, creating the potential for multi‐state or multi‐country clusters [44]. These findings raise concerns regarding urban zoonotic surveillance and emphasize that hantavirus infection is no longer confined to remote rural environments. Public health authorities in urban centres must now consider hantavirus in the differential diagnosis of febrile illness with renal or pulmonary manifestations, particularly in patients with reported rodent exposure, including pet rats.

In Italy, hantavirus disease remains poorly characterized and likely underrecognized. Although autochthonous cases are infrequently reported, with only sporadic cases documented in the literature, serological and ecological evidence suggests that circulation of orthohantaviruses is broader than generally appreciated. Serosurveys in forestry workers, hunters, and military personnel have detected antibodies against PUUV, DOBV, and SEOV, indicating past exposure. The combination of ecological suitability (extensive forested areas inhabited by bank voles and yellow‐necked mice), expanding rodent habitats due to land abandonment and reforestation, climate variability that may favour rodent population growth, and increased awareness among clinicians may contribute to improved recognition of cases in Southern Europe over the coming years. Italy, like other Mediterranean countries, may face increasing detection of hantavirus infections as diagnostic testing becomes more widely available [8, 37, 41, 43, 46, 47].

Reservoir ecology remains central to understanding hantavirus persistence and spillover. Rodent abundance, species diversity, interspecies interactions, habitat fragmentation, and the presence of alternative prey for predators all influence viral transmission dynamics within reservoir populations and the risk of spillover to humans. Recent studies suggest that multiple rodent species may participate in maintenance cycles for some pathogenic hantaviruses, potentially complicating traditional assumptions regarding strict host specificity. For example, SNV has been detected in several rodent species beyond its primary reservoir, *P. maniculatus*, suggesting that maintenance may involve multi‐host communities rather than single species [42, 49, 67, 68]. Spillover into atypical hosts, including insectivores, bats, and even domestic animals, has also been documented, raising additional questions regarding viral adaptation, ecological plasticity, and the potential for novel transmission pathways [68].

Human exposure is strongly associated with occupational and environmental risk factors including forestry work (timber harvesting, brush clearing), farming (especially activities involving hay or grain storage), military activities (field exercises, bivouacking), rodent‐infested dwellings (peridomestic exposure in rural and peri‐urban homes), and outdoor recreation (camping, hiking, hunting). Military populations represent a historically important risk group because of prolonged field exposure, close interaction with reservoir habitats, and the potential for person‐to‐person transmission in congregate settings. Outbreaks among military personnel have been documented in several countries, including the Balkans, South Korea, and Germany [8, 69, 70]. Land‐use change, deforestation for agriculture or urban development, agricultural expansion into forested areas, and peri‐urban development at the wildland‐urban interface may further increase opportunities for rodent‐human contact by bringing humans and rodents into closer proximity [11].

**FIGURE 1:**
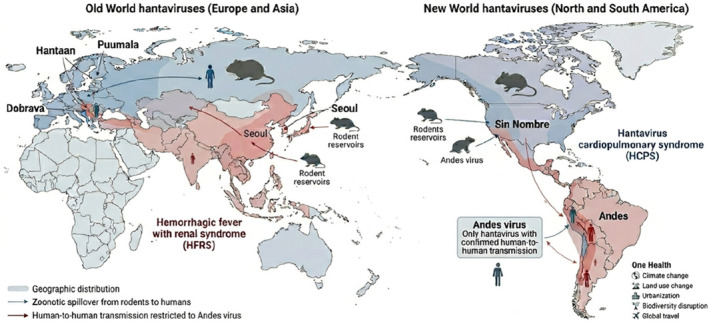
Global epidemiology, reservoirs, and transmission pathways of hantaviruses. Geographic distribution of major old world and new world hantaviruses, including their principal rodent reservoirs, associated clinical syndromes, and dominant transmission pathways [1, 5, 48, 49]. Old world hantaviruses are primarily associated with haemorrhagic fever with renal syndrome (HFRS), whereas new world hantaviruses predominantly cause hantavirus cardiopulmonary syndrome (HCPS) [1, 5]. Andes virus remains the only hantavirus with confirmed human‐to‐human transmission [11, 13]. Environmental and ecological drivers potentially influencing hantavirus emergence, including climate change, land‐use modification, urbanisation, biodiversity disruption, and global mobility, are also highlighted [2, 35, 37, 43].

Collectively, these observations support the view that hantavirus emergence is increasingly driven by environmental disruption and global ecological change. Climate change is altering the geographic distribution of reservoir hosts, with some species shifting their ranges poleward. Biodiversity loss, particularly the decline of rodent predators such as foxes, raptors, and snakes, may remove natural checks on rodent populations, leading to higher densities and increased viral circulation. Land‐use change, including reforestation of abandoned agricultural land, can create new habitats for reservoir species. Future surveillance strategies will likely require integration of climate science (temperature, precipitation, vegetation indices), wildlife ecology (rodent trapping, serosurveys, predator abundance), genomic surveillance (viral sequencing, phylodynamics), and human epidemiology within coordinated One Health frameworks. The global distribution, reservoir hosts, and major transmission pathways of clinically relevant hantaviruses are illustrated in Figure 1.

## 4 Outbreak Investigation, Contact Tracing, and Transmission Dynamics

The recognition of person‐to‐person transmission in Andes virus (ANDV) infection has fundamentally transformed outbreak investigation strategies for hantavirus disease, distinguishing ANDV from all other known hantaviruses. Unlike classical rodent‐borne exposure events, where risk is primarily environmental and linked to aerosolised excreta from infected rodents, Andes virus outbreaks frequently require detailed reconstruction of interpersonal interactions, temporal exposure windows, household dynamics, caregiving networks, and healthcare‐associated contacts. This paradigm shift has necessitated the adaptation of contact tracing methodologies traditionally used for respiratory viruses such as influenza, SARS‐CoV‐2, and Ebola virus to the context of a zoonotic pathogen that retains its ecological reservoir [11, 12].

Household clusters remain the most characteristic epidemiological pattern associated with interhuman transmission of Andes virus. Investigations performed during outbreaks in Chile and Argentina demonstrated that secondary infections occurred predominantly among close household contacts and intimate partners exposed during the prodromal phase of disease, often before the index patient developed severe cardiopulmonary manifestations requiring hospitalisation. In several well‐documented clusters, secondary cases developed 15–25 days after exposure to the index case, a timeline compatible with the known incubation period of hantavirus infection and inconsistent with simultaneous rodent exposure. These observations were particularly important because they challenged the long‐standing assumption that all clustered cases merely reflected shared environmental exposure rather than true human‐to‐human transmission [11, 12, 13].

Distinguishing common‐source environmental exposure from true human transmission remains methodologically complex, especially in rural endemic regions where household members may share similar occupational, recreational, and domestic environments, including exposure to rodent habitats. For this reason, detailed exposure histories, including activities such as cleaning barns, sweeping cabins, camping, farming, and firewood collection, combined with temporal sequencing of symptom onset and molecular epidemiology are central components of modern hantavirus outbreak investigation. In several outbreaks, epidemiological links combined with incubation period calculations and phylogenetic analysis of viral sequences from index and secondary cases strongly supported secondary transmission chains that were incompatible with isolated rodent exposure. The use of whole‐genome sequencing has further refined these investigations by providing the resolution needed to distinguish between independent spillover events from a common rodent source and true person‐to‐person transmission [11, 12].

Serial interval analysis, the time between symptom onset in an index case and symptom onset in a secondary case, has further improved understanding of transmission dynamics. Available evidence suggests that infectivity is greatest during the late prodromal and early cardiopulmonary phases, a period lasting approximately 2–5 days, coinciding with increasing viraemia and viral shedding in respiratory secretions and saliva. Peak viral loads, as measured by quantitative reverse transcription polymerase chain reaction (qRT‐PCR), have been documented during this window, often before patients meet clinical criteria for hospitalisation. The demonstration of detectable viral RNA in saliva and upper respiratory samples, including oropharyngeal swabs and sputum, provided a biologically plausible explanation for the temporal clustering observed among close contacts, supporting transmission via respiratory droplets or direct contact with contaminated saliva. Notably, viral shedding has been documented to persist for several days after symptom onset, extending the potential window for transmission [13, 15].

Identification of high‐risk contacts therefore represents a critical component of public health management during Andes virus outbreaks. Close household exposure, defined as sleeping in the same room, sharing meals, or providing direct care, appears to confer the greatest risk, with secondary attack rates in some clusters exceeding 10%–15%. Prolonged face‐to‐face interaction (greater than several hours), caregiving activities (bathing, feeding, assisting with mobility), and direct exposure to respiratory secretions (such as during coughing episodes or aerosol‐generating procedures) similarly elevate risk. By contrast, brief social interactions, casual community exposure (such as sharing a workplace without close contact), and transient encounters have not been consistently associated with secondary transmission. Public health authorities in endemic regions have therefore developed protocols for active surveillance of high‐risk contacts, including daily temperature monitoring and symptom screening for up to 21–28 days following last exposure, with low‐threshold diagnostic testing for those who develop fever, myalgia, or respiratory symptoms [11, 12].

Concerns regarding healthcare‐associated transmission have also shaped infection prevention policies. Although nosocomial spread has been documented in some outbreak investigations, particularly involving unprotected exposure during intubation, aerosol‐generating procedures, or handling of infected body fluids, available evidence overall suggests that transmission to healthcare workers is uncommon when standard precautions are appropriately implemented. Serological investigations among healthcare personnel exposed to patients with HCPS failed to demonstrate widespread nosocomial infection, supporting the effectiveness of infection prevention measures in clinical settings, including hand hygiene, personal protective equipment (PPE), and respiratory hygiene. Nevertheless, rare cases of healthcare‐associated transmission have been reported, underscoring the need for vigilance [12, 71]. Current recommendations from the Centres for Disease Control and Prevention and the World Health Organization generally support the use of standard and droplet precautions for suspected Andes virus infection, with escalation to airborne precautions during aerosol‐generating procedures such as intubation, bronchoscopy, and non‐invasive ventilation [72, 73].

Another unresolved question concerns the potential for transmission heterogeneity and superspreading events. Although large explosive outbreaks have not been typical of Andes virus epidemiology, most clusters involve 2–5 cases, some reports suggest that transmission efficiency may vary substantially between individuals. Viral load (which may be influenced by host immune status and viral genetics), host immune responses (including inflammatory phenotype and magnitude of cytokine response), symptom severity (cough intensity and frequency), behavioural factors (mask use, hygiene practices, proximity during interactions), and environmental conditions (ventilation, crowding, humidity) may all influence secondary attack rates. Whether specific viral genetic determinants, such as polymorphisms in the glycoprotein genes that enhance stability or receptor affinity, favour enhanced transmissibility remains unknown and represents an active area of investigation [11, 13].

The increasing interconnectedness of global travel has introduced additional operational challenges for outbreak investigation and contact tracing. The recent World Health Organization report describing a multinational hantavirus cluster linked to cruise ship travel highlighted the difficulties associated with international contact tracing, passenger notification across multiple jurisdictions, cross‐border surveillance coordination, and public communication. In this event, cases were identified in passengers from several countries who had shared common spaces, dining areas, and excursion activities over a period of days. Although widespread transmission did not occur, the event demonstrated how rapidly a traditionally localised zoonotic infection can generate multinational public health concern, requiring coordination between national health authorities, international shipping regulators, and WHO regional offices. These experiences have reinforced the need for standardized protocols for travel‐related hantavirus exposure, including passenger notification systems, cross‐border data sharing agreements, and harmonised case definitions [16].

**FIGURE 2:**
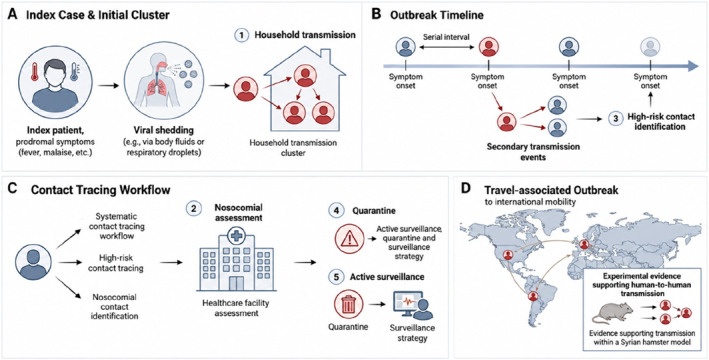
Human‐to‐human transmission and outbreak investigation of Andes virus. Schematic representation of the principal epidemiological and public health features associated with confirmed person‐to‐person transmission of andes virus [11, 12, 13]. (A) Index case and initial cluster: household transmission cluster originating from an index patient with prodromal symptoms and viral shedding via body fluids or respiratory droplets [11, 12, 13]. (B) Outbreak timeline: sequential phases including household transmission with symptom onset dates, nosocomial assessment with active surveillance, quarantine strategies, and high‐risk contact identification leading to secondary transmission events [11, 12, 13]. (C) Contact tracing workflow: systematic identification of high‐risk and nosocomial contacts, healthcare facility assessment, active surveillance, and implementation of surveillance strategies [11, 12, 71, 72]. (D) Travel‐associated outbreak and international mobility: challenges posed by travel‐related clusters in the context of increasing global mobility [16]. Experimental evidence supporting Andes virus transmissibility in animal models, including transmission within a Syrian hamster model, is also highlighted [14, 15]. Healthcare‐associated exposure assessment and infection prevention measures are informed by standard precaution guidelines [71, 72].

Figure 2 provides a schematic representation of the principal epidemiological and public health features associated with confirmed person‐to‐person transmission of Andes virus, including household transmission clusters, serial interval analysis, high‐risk contact identification, healthcare‐associated exposure assessment, quarantine and surveillance strategies, and the challenges posed by travel‐associated outbreaks.

## 5 Clinical Manifestations, Immunopathogenesis, Diagnostics, and Management

Hantavirus infections encompass a broad clinical spectrum ranging from asymptomatic seroconversion to fulminant multiorgan failure. Disease severity varies substantially according to viral species, host factors (including age, comorbidities, and genetic background), immune responses, and timing of clinical recognition. Incubation periods typically range from one to 5 weeks, with an average of two to 4 weeks, depending on the viral species and inoculum size. This section integrates the clinical presentation, underlying immunopathogenesis, diagnostic approaches, surveillance strategies, and current management of hantavirus disease [1, 6, 7, 9, 50].

### 5.1 Clinical Syndromes: HFRS and HCPS

Traditionally, hantavirus disease has been divided into two major clinical syndromes: haemorrhagic fever with renal syndrome (HFRS) in Eurasia and hantavirus cardiopulmonary syndrome (HCPS) in the Americas. Although useful epidemiologically, this distinction oversimplifies the overlapping pathophysiology shared across hantavirus infections. Both syndromes are characterised by endothelial dysfunction, increased vascular permeability, thrombocytopaenia, and variable involvement of pulmonary, renal, and cardiovascular systems. The clinical presentation reflects the predominant vascular bed affected: renal microvasculature in HFRS and pulmonary microvasculature in HCPS, though cross‐over manifestations are increasingly recognized [1, 6, 74].

#### 5.1.1 Renal Manifestations and Disease Spectrum

HFRS is most commonly caused by Hantaan virus (HTNV), Dobrava‐Belgrade virus (DOBV), Seoul virus (SEOV), and Puumala virus (PUUV). Clinical severity ranges from mild nephropathia epidemica associated with Puumala virus, often presenting as influenza‐like illness with mild renal impairment, to severe haemorrhagic disease with shock, profound thrombocytopaenia, and acute kidney injury requiring renal replacement therapy caused by Hantaan virus. Dobrava‐Belgrade virus occupies an intermediate position, with severity varying by genotype [6, 7, 8].

After an incubation period typically ranging from 2 to 4 weeks, patients develop abrupt onset of fever (often > 39°C), severe headache (retro‐orbital or frontal), myalgia (particularly lumbosacral back pain), malaise, anorexia, nausea, vomiting, and abdominal pain. Conjunctival injection (suffusion), pharyngeal injection, palatal petechiae, transient visual disturbances, and thrombocytopaenia (often < 100,000/μL) may occur early [6, 8]. Classical descriptions divide HFRS into five sequential phases, though many patients, particularly those with milder disease, do not progress through all stages.

- Febrile phase (3–7 days): High fever, headache, myalgia, flushing, and conjunctival injection. Thrombocytopaenia and proteinuria become evident.
- Hypotensive phase (hours to 2 days): Sudden defervescence may coincide with hypotension, shock, and haemorrhagic manifestations (ecchymoses, gastrointestinal bleeding). More common in severe HTNV and DOBV infections.
- Oliguric phase (3–7 days): Decreasing urine output (< 400 mL/day), rising creatinine, hyperkalemia, metabolic acidosis. Dialysis may be required.
- Polyuric phase (days to weeks): Diuresis of up to 3–6 L/day, with risk of hypovolaemia and electrolyte disturbances.
- Convalescent phase (weeks to months): Gradual recovery of renal function.

Renal manifestations and outcomes. Acute kidney injury (AKI) is the central clinical feature, ranging from mild creatinine elevation to severe renal failure requiring dialysis. Proteinuria is nearly universal and correlates with disease severity. Coagulopathy, thrombocytopaenia, endothelial activation, and haemorrhagic manifestations reflect widespread vascular dysfunction [60, 75]. Biomarker studies have identified associations between severe disease and elevated inflammatory mediators (IL‐6, TNF‐α), endothelial injury markers (soluble thrombomodulin, von Willebrand factor), and coagulation abnormalities (elevated D‐dimers) [59, 61, 75, 76, 77]. Although mortality for PUUV is generally low (< 1%), severe disease can occur in older individuals and those with comorbidities. Long‐term follow‐up studies extending up to 20 years suggest that some patients experience persistent renal abnormalities, chronic fatigue, and reduced quality of life [9, 78].

#### 5.1.2 Capillary Leak Syndrome and Cardiopulmonary Failure

HCPS is predominantly associated with New World hantaviruses including Andes virus (ANDV) and Sin Nombre virus (SNV) and remains one of the most severe viral zoonoses in the Americas, with reported case fatality rates of 30%–40%. Andes virus is unique among hantaviruses in its ability to transmit person‐to‐person, while Sin Nombre virus is the most common cause of HCPS in North America [1, 66, 79].

The prodromal phase (2–7 days) is characterised by fever, myalgia (often severe in the lower back and thighs), headache, gastrointestinal symptoms, and nonspecific influenza‐like manifestations. This phase is indistinguishable from many other viral illnesses, contributing to diagnostic delays. This is followed by abrupt cardiopulmonary deterioration with rapidly progressive dyspnoea, tachypnoea, hypoxaemia (often refractory to supplemental oxygen), non‐cardiogenic pulmonary oedema, and shock resulting from capillary leak and myocardial dysfunction. The transition from prodrome to respiratory failure can occur within hours [1, 79].

Profound vascular permeability rather than direct viral cytotoxicity drives the pulmonary manifestations. Patients frequently develop haemoconcentration (due to plasma extravasation), severe thrombocytopaenia (< 50,000/μL), elevated lactate levels, and hypotension requiring vasopressor support. The haemodynamic profile is typically characterised by low cardiac output and high systemic vascular resistance, distinguishing HCPS from distributive shock seen in sepsis [23, 56, 57, 58, 59, 60, 79]. Meta‐analyses have identified independent prognostic markers for fatal outcomes, including shock at presentation, severe thrombocytopaenia, elevated lactate (> 4 mmol/L), renal dysfunction, extensive pulmonary involvement, and need for mechanical ventilation. Proteinuria additionally represents an early marker of severe disease [10, 50].

Survivors of severe HCPS often experience prolonged recovery. Recent studies have highlighted persistent fatigue, dyspnoea on exertion, neurocognitive symptoms, respiratory limitations, and impaired quality of life months to years after acute infection, particularly among patients requiring extracorporeal membrane oxygenation (ECMO). Some survivors report post‐traumatic stress symptoms related to their critical illness [80].

Increasing evidence suggests that renal abnormalities are common in HCPS and that pulmonary involvement may occur in severe Old World infections, reinforcing the concept that hantavirus disease exists along a clinicopathological continuum of endothelial dysfunction rather than as entirely separate syndromes [1, 6, 9].

### 5.2 Immunopathogenesis and Endothelial Dysfunction

Despite major advances in hantavirus biology, the precise mechanisms responsible for severe disease remain incompletely understood. A defining feature across both HFRS and HCPS is profound endothelial dysfunction leading to increased vascular permeability, tissue oedema, hypotension, and organ injury. Importantly, this process occurs in the absence of widespread endothelial destruction, suggesting that immune‐mediated dysregulation rather than direct cytopathic damage represents the principal driver of disease severity [17, 18, 19, 20, 21, 23, 56, 57, 58, 59, 60].

Endothelial cells constitute the primary target of hantavirus infection in humans. Following viral entry, pathogenic hantaviruses establish productive infection while largely preserving endothelial cell viability. This ability to maintain infected endothelial networks without extensive cell death may contribute to sustained vascular dysregulation and prolonged inflammatory activation [23, 54, 60].

Several molecular pathways have been implicated in capillary leak. Experimental studies demonstrated that hantaviruses sensitise endothelial cells to vascular endothelial growth factor (VEGF), thereby amplifying permeability responses and disrupting barrier integrity [60]. Additional work identified activation of Src family kinases (which destabilise intercellular junctions), TLR4‐TRAF6 signalling pathways, and RhoA‐mediated cytoskeletal rearrangements (which promote stress fibre formation and intercellular gaps) as key contributors to endothelial hyperpermeability [56, 57, 60].

Pericyte infection may further amplify vascular injury. Pericytes are contractile cells that surround endothelial cells and are essential for microvascular stability. Andes virus has been shown to infect human pericytes and induce signalling cascades that increase endothelial permeability, supporting the concept that vascular dysfunction results from complex multicellular interactions within the microvascular environment rather than isolated endothelial abnormalities alone [23].

Host immune responses play a central role in disease progression. Although robust antiviral immunity is essential for viral control, excessive or dysregulated immune activation contributes substantially to tissue injury and vascular leakage. Elevated concentrations of pro‐inflammatory cytokines including IL‐6, TNF‐α, IFN‐γ, and chemokines have consistently been associated with severe disease [61, 81]. Recent studies have particularly emphasised the importance of IL‐6 *trans*‐signalling pathways in mediating endothelial barrier dysfunction during hantavirus infection. Increased IL‐6 *trans*‐signalling correlates with disease severity, capillary leak, and clinical deterioration, identifying this pathway as a potential therapeutic target [61].

Activated CD8‐positive T cells accumulate during acute infection and may contribute to endothelial damage through bystander activation and cytotoxic signalling. Experimental evidence suggests that IL‐15‐driven activation of cytotoxic lymphocytes may promote endothelial injury through NKG2D‐dependent pathways. At the same time, delayed viral clearance despite marked T‐cell activation has been documented in severe HCPS, suggesting that excessive immune activation does not necessarily translate into effective viral control [58, 82, 83].

Hantaviruses possess multiple mechanisms to modulate interferon signalling and dampen early antiviral responses [20, 21, 22]. Comparative studies between reservoir hosts and humans indicate that rodent reservoirs maintain tightly regulated antiviral responses that limit immunopathology while allowing persistent infection. In contrast, human infection appears characterised by exaggerated inflammatory activation and dysregulated endothelial signalling [24, 52, 53, 55]. Activation of innate lymphoid cells has also been demonstrated during HFRS, suggesting that previously underappreciated immune pathways may contribute to both protective immunity and pathological inflammation [84].

Coagulation abnormalities represent another major component of hantavirus pathogenesis. Thrombocytopaenia, platelet dysfunction, endothelial activation, and disturbances of coagulation pathways are common in severe disease [59, 76, 77]. Elevated soluble thrombomodulin levels, markers of endothelial injury, and evidence of microvascular coagulation activation have all been associated with poor outcomes [59]. Major biomarkers and prognostic indicators associated with severe hantavirus disease are summarised in Table 2.

**TABLE 2:** Biomarkers and prognostic indicators associated with severe hantavirus disease.

Biomarker/parameter	Proposed mechanism	Association with severe disease
Thrombocytopaenia	Endothelial activation and platelet consumption	Increased risk of haemorrhage and severe capillary leak
Proteinuria	Glomerular endothelial injury	Associated with mortality and severe HCPS
Elevated IL‐6	Hyperinflammatory response	Correlates with vascular leakage and disease severity
Soluble thrombomodulin [sTM)	Endothelial dysfunction	Predictor of poor prognosis in HFRS
Elevated VEGF signalling	Increased vascular permeability	Associated with pulmonary oedema and shock
High viral load/prolonged viraemia	Persistent viral replication	Associated with severe disease and transmission potential
Activated CD8+ T cells	Immunopathological endothelial damage	Correlates with severe HFRS
Delayed viral clearance	Persistent immune activation	Associated with worse clinical outcomes
Elevated creatinine	Acute kidney injury	Marker of severe renal disease
Hypoalbuminemia	Capillary leak syndrome	Associated with critical illness
Need for ECMO or vasopressors	Cardiopulmonary failure	Strong predictor of mortality

*Note:* Biomarkers associated with endothelial dysfunction, immune activation, coagulation abnormalities, and capillary leak may contribute to risk stratification in severe hantavirus disease. Endothelial dysfunction markers include VEGF signalling [60] and pericyte infection [23]. Immune activation markers include elevated IL‐6 levels [61], activated CD8+ T cells [82, 83], and cytokine dysregulation [81]. Coagulation abnormalities and endothelial injury markers include thrombocytopaenia, soluble thrombomodulin [59], and proteinuria [10]. Capillary leak and disease severity indicators include elevated creatinine, hypoalbuminemia, high viral load with prolonged viraemia [13], delayed viral clearance [58], and need for ECMO or vasopressors [75]. These biomarkers are associated with disease severity in both HFRS and HCPS [56, 57, 74, 76].

**FIGURE 3:**
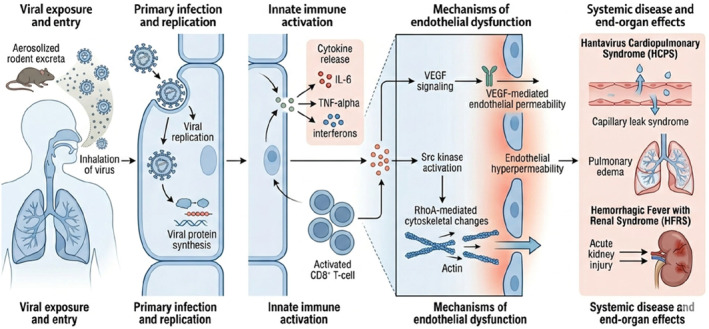
Pathogenesis of hantavirus disease and mechanisms of endothelial dysfunction. Schematic overview of the major mechanisms involved in hantavirus pathogenesis, including viral entry through inhalation of aerosolised rodent excreta, endothelial infection, innate and adaptive immune activation, cytokine dysregulation, and endothelial barrier disruption [17, 19, 20, 21, 22, 23]. VEGF signalling, Src kinase activation, and RhoA‐mediated cytoskeletal rearrangements contribute to endothelial hyperpermeability and capillary leak [56, 57, 60], while IL‐6 *trans*‐signalling mediates cytokine secretion and barrier dysfunction [61]. Activated CD8+ T cells may contribute to endothelial injury through bystander activation and NKG2D‐dependent pathways [82, 83], and innate lymphoid cells are activated in HFRS with modulation by hantavirus‐induced type I interferons [84]. These mechanisms lead to systemic manifestations including pulmonary oedema in HCPS and acute kidney injury in HFRS.

Disease expression differs substantially between hantavirus species. New World hantaviruses generally induce more severe pulmonary capillary leak and cardiopulmonary collapse, whereas Old World hantaviruses more commonly target renal microvascular beds. Differential tissue tropism, receptor usage, immune activation patterns, and endothelial signalling pathways may all contribute to these phenotypic differences [1, 6, 7, 32, 52, 53, 54]. Age, comorbidities, viral inoculum, genetic background, and baseline immune status likely influence disease severity, although robust predictive models remain limited [9, 50, 78]. The principal mechanisms underlying hantavirus‐associated endothelial dysfunction and systemic disease are illustrated in Figure 3.

### 5.3 Diagnostics

Early diagnosis of hantavirus infection remains challenging because initial clinical manifestations are frequently nonspecific and may resemble influenza, leptospirosis, dengue, rickettsial infections, viral hepatitis, bacterial sepsis, or other causes of febrile illness with thrombocytopaenia. Delayed recognition may significantly worsen outcomes, particularly in HCPS where clinical deterioration can occur abruptly over a matter of hours [1, 4, 6, 8].

Serological testing remains the cornerstone of diagnosis in most settings. Detection of hantavirus‐specific IgM antibodies typically confirms acute infection, while rising IgG titres support recent exposure, most commonly by enzyme‐linked immunosorbent assay (ELISA) or indirect immunofluorescence; commercially available lateral flow immunochromatographic assays also provide rapid, easy‐to‐implement point‐of‐care detection of hantavirus‐specific antibodies, particularly useful in settings without immediate access to a reference laboratory [4, 85]. In HCPS, early serological responses may additionally correlate with clinical outcomes and disease evolution [86].

Reverse transcription polymerase chain reaction (RT‐PCR) assays allow detection of viral RNA in blood, respiratory secretions, and tissue samples before full seroconversion occurs. Molecular approaches are especially valuable for Andes virus because they may support contact tracing efforts and identification of transmission chains [4, 13, 85]. Recent prospective studies demonstrated prolonged viraemia and viral shedding during acute Andes virus infection, expanding the potential utility of serial molecular monitoring in selected patients [13]. Improved molecular characterisation additionally facilitates phylogenetic analysis during outbreak investigations and supports genomic surveillance efforts [85].

Advances in structural virology and immunology are contributing to the development of next‐generation diagnostic tools. Multiplex serological assays, high‐throughput sequencing approaches, and broader orthobunyavirus surveillance platforms may improve recognition of atypical or previously underdiagnosed infections [4].

Important diagnostic limitations persist. Many endemic regions face restricted laboratory access, delayed sample processing, and limited surveillance capacity. Underdiagnosis remains particularly problematic in low‐resource settings and in regions where hantavirus infection is not routinely considered within differential diagnoses [4, 47]. Surveillance systems vary substantially between countries. While some regions maintain structured hantavirus registries and integrated surveillance networks, others rely primarily on passive reporting mechanisms [87]. The development of registries such as HantaReg represents an important step towards harmonised clinical data collection, epidemiological monitoring, and collaborative research [87].

The growing ecological complexity of hantavirus circulation further emphasises the need for integrated surveillance strategies combining human, animal, and environmental data. Rodent monitoring programs, genomic surveillance, climate modelling, and early warning systems may improve outbreak prediction and preparedness [37, 38, 39, 40]. In Europe, increasing awareness of Seoul virus circulation and evidence of underrecognized hantavirus activity in Southern Europe support the need for broader diagnostic consideration and improved surveillance frameworks [43, 44, 47]. Similar challenges exist in Latin America, where ecological diversity and limited access to specialised diagnostics likely contribute to substantial underestimation of disease burden. Future diagnostic strategies will likely depend increasingly on rapid molecular testing, point‐of‐care platforms, genomic epidemiology, and integrated One Health surveillance [37, 38, 87].

### 5.4 Current Management Strategies and Critical Care

Management of hantavirus infection remains predominantly supportive because no antiviral therapy has yet demonstrated consistent clinical efficacy across the spectrum of disease. Early recognition, rapid haemodynamic stabilisation, intensive monitoring, and timely escalation of organ support therefore remain central determinants of survival, particularly in HCPS [1, 6, 79, 88].

Management of HFRS focuses primarily on fluid balance optimization, correction of electrolyte disturbances, haemodynamic support, and management of acute kidney injury. Excessive fluid administration may worsen capillary leak and pulmonary oedema, whereas inadequate resuscitation can aggravate shock and renal hypoperfusion. Careful individualised volume management is therefore essential. Renal replacement therapy may be required in severe cases complicated by oliguric renal failure, metabolic acidosis, or refractory electrolyte abnormalities. Most patients ultimately recover renal function, although prolonged convalescence and residual renal impairment may occur in selected individuals [6, 78]. Coagulopathy and thrombocytopaenia frequently complicate severe HFRS. Management remains largely supportive and may include transfusion support in cases of clinically significant bleeding or invasive procedures [74, 76].

HCPS remains one of the most challenging viral syndromes encountered in critical care medicine because of its rapid progression and profound cardiopulmonary instability [1, 79, 88]. The transition from prodromal symptoms to fulminant respiratory failure may occur within hours. Patients frequently require aggressive oxygen support, invasive mechanical ventilation, vasopressor therapy, and advanced haemodynamic monitoring. Pulmonary oedema results primarily from capillary leak rather than volume overload, and management therefore requires careful balancing of tissue perfusion against worsening respiratory compromise.

ECMO has emerged as a potentially lifesaving intervention in refractory HCPS with severe hypoxaemia or circulatory collapse [79, 80]. Several observational studies suggest that early referral to experienced ECMO centres may improve survival among critically ill patients, particularly younger individuals without advanced comorbidities. Alternative extracorporeal strategies have also been explored. Targeted high‐volume hemofiltration has been proposed as a potential approach capable of modulating inflammatory responses and reducing the need for ECMO in selected patients, although evidence remains limited [89].

Critical care transport itself represents a major logistical challenge in endemic rural regions where access to tertiary intensive care units may be delayed. Specialised transport systems, rapid recognition protocols, and regional coordination pathways therefore remain essential components of preparedness planning [88].

### 5.5 Antiviral and Immunomodulatory Therapies, Vaccines, and Emerging Countermeasures

To date, no specific antiviral therapy has become standard of care for hantavirus disease. Ribavirin demonstrated some benefit in earlier studies of HFRS, particularly when administered early during infection, but evidence remains inconsistent and less convincing for HCPS [1, 6, 8].

Growing understanding of hantavirus immunopathogenesis has stimulated increasing interest in host‐directed therapies aimed at reducing endothelial dysfunction and excessive inflammatory activation. However, clinical evidence supporting corticosteroids, cytokine‐directed therapies, or immunomodulators remains limited. Candidate approaches targeting VEGF signalling, Src kinase activation, RhoA pathways, and IL‐6 *trans*‐signalling are under investigation, but none have yet been validated in clinical trials [57, 60, 61].

Monoclonal antibody therapies represent one of the most promising translational developments. Experimental studies demonstrated that human monoclonal antibodies targeting hantavirus glycoproteins can provide protection in animal models, including post‐exposure settings [29, 30, 31]. Structural analyses additionally identified broadly neutralising antibodies capable of recognising conserved quaternary epitopes shared across multiple hantavirus species, raising the possibility of pan‐hantavirus therapeutic approaches [28, 30, 31, 90]. At present, however, these strategies remain investigational and require validation in human clinical trials.

The absence of licenced broadly protective vaccines remains among the most important unmet needs. Historically, inactivated vaccines against Hantaan virus were developed and used in some Asian countries, but concerns persisted regarding durability of protection, variable immunogenicity, and limited cross‐protection [25, 26]. More recently, attention has shifted towards next‐generation vaccine technologies, including DNA vaccines, mRNA platforms, glycoprotein‐based vaccines, and prefusion‐stabilised glycoprotein antigens [25, 27, 33, 34]. These platforms aim to induce broader and more potent neutralising immune responses by targeting the Gn and Gc surface glycoproteins, which contain the principal neutralising epitopes.

**FIGURE 4:**
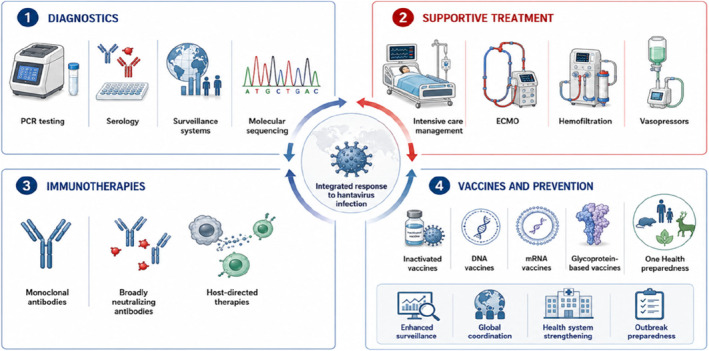
Current and emerging strategies against hantavirus infection. Overview of current and emerging approaches for the diagnosis, management, prevention, and translational treatment of hantavirus disease. The figure summarises advances in molecular diagnostics and surveillance systems [4, 13], supportive critical care strategies including extracorporeal membrane oxygenation (ECMO) and hemofiltration [79, 88, 89], monoclonal antibody‐based immunotherapies [29, 30, 31], host‐directed therapeutic approaches targeting endothelial dysfunction and inflammatory pathways [57, 60, 61], and next‐generation vaccine platforms including DNA, mRNA, and glycoprotein‐based vaccines [25, 27, 33, 34]. Integrated One Health preparedness strategies incorporating surveillance, health system strengthening, and coordinated outbreak response are also highlighted [37, 38, 87].

Effective preparedness for hantavirus emergence requires integrated One Health frameworks capable of combining wildlife surveillance, climate science, molecular epidemiology, critical care preparedness, and coordinated international public health response [37, 38, 45, 62]. The convergence of structural biology, monoclonal antibody engineering, mRNA vaccine technology, and One Health surveillance now places hantavirus research in a markedly different position than a decade ago. Current diagnostic, therapeutic, immunological, and preventive strategies against hantavirus infection are summarised in Figure 4 and Table 3.

**TABLE 3:** Vaccines and emerging therapeutic strategies against hantavirus infection.

Strategy	Platform/approach	Target	Development stage	Main findings
Inactivated vaccines	Whole‐virus vaccines	HTNV, SEOV	Licenced in parts of asia	Partial protection with variable immunogenicity
DNA vaccines	Plasmid‐based vaccines	HTNV, ANDV	Clinical development	Induction of neutralising antibodies
mRNA vaccines	Modified nucleoside mRNA platforms	ANDV, HTNV	Preclinical	Strong humoural responses and improved immunogenicity
Glycoprotein‐based vaccines	Recombinant glycoprotein constructs	Gn/Gc proteins	Preclinical	Broad neutralising antibody induction
Prefusion‐stabilised glycoprotein vaccines	Stabilised prefusion glycoprotein antigens	HTNV glycoproteins	Experimental	Enhanced germinal centre activation and antibody maturation
Monoclonal antibodies	Human neutralising antibodies	Multiple hantaviruses	Preclinical/experimental	Cross‐protective neutralisation against diverse hantaviruses
Pan‐hantavirus antibodies	Broadly neutralising antibodies	Conserved quaternary epitopes	Experimental	Protection in animal models
Current advanced supportive care	ECMO, hemofiltration, vasopressors	Severe HCPS	Clinical implementation	Improved survival in fulminant disease
Host‐directed therapies	Immune and endothelial modulation	Vascular permeability pathways	Experimental	Potential reduction of capillary leak and endothelial dysfunction
One health surveillance strategies	Integrated ecological and genomic surveillance	Reservoir and spillover monitoring	Public health implementation	Early outbreak detection and preparedness

*Note:* Recent advances in structural virology, immunology, and translational medicine are accelerating the development of novel vaccine platforms, including inactivated vaccines, DNA vaccines, mRNA platforms, glycoprotein‐based vaccines, and prefusion‐stabilised glycoprotein antigens [25, 26, 27, 33, 34]. Monoclonal antibody strategies have yielded human neutralising antibodies capable of cross‐protective neutralisation against diverse hantaviruses, including pan‐hantavirus antibodies targeting conserved quaternary epitopes [28, 29, 30, 31]. Host‐directed therapies targeting endothelial dysfunction and inflammatory pathways, including modulation of VEGF signalling, Src kinase, and IL‐6 *trans*‐signalling, are under investigation [57, 60, 61]. Current advanced supportive care includes ECMO, hemofiltration, and vasopressor support for severe HCPS [79, 89]. Integrated One Health surveillance strategies combining ecological and genomic surveillance for reservoir and spillover monitoring are increasingly recognized as essential for early outbreak detection and preparedness [37, 38, 45, 62].

## 6 Conclusions

Hantaviruses continue to evolve from geographically restricted zoonotic pathogens into increasingly complex global health threats shaped by ecological disruption, climate variability, urbanisation, and changing human‐animal interactions. Recent advances have substantially transformed understanding of hantavirus biology and disease. The confirmation of person‐to‐person transmission for Andes virus, growing evidence of climate‐sensitive transmission dynamics, improved characterisation of endothelial dysfunction, and rapid progress in vaccine and monoclonal antibody development collectively mark a new phase in hantavirus research. At the same time, important challenges persist: diagnostic delays remain common, therapeutic options are limited, and surveillance systems vary substantially across regions. Europe, including Southern European countries such as Italy, may face increasing recognition of previously underdiagnosed hantavirus circulation in coming years [1, 2, 4, 8, 37, 41, 43, 47].

Despite important advances, major gaps persist across epidemiology, pathogenesis, diagnostics, therapeutics, and public health preparedness. One of the most consequential unresolved questions concerns the determinants of person‐to‐person transmission. Andes virus remains unique among hantaviruses in its demonstrated ability to spread between humans, yet the precise viral and host factors responsible for this property remain poorly understood. Human‐to‐human transmission appears to be largely restricted to Andes virus and has been documented primarily in household and close‐contact settings, with household clusters and prolonged close exposure representing the most consistently identified epidemiological risk factors for secondary transmission. Viral shedding during the prodromal phase raises concern for transmission before the onset of severe cardiopulmonary manifestations, complicating outbreak containment strategies. Although current evidence does not support sustained airborne community transmission, respiratory secretions and salivary exposure are considered plausible routes of spread. Nosocomial transmission appears uncommon when standard infection prevention and control precautions are implemented appropriately. Rapid identification and monitoring of high‐risk contacts are essential to limit secondary transmission during outbreaks, and quarantine measures with active surveillance may be considered in selected high‐risk exposure settings, particularly during confirmed Andes virus clusters [11, 12, 13, 14, 15, 16, 72, 91]. Whether additional hantaviruses could eventually acquire similar transmissibility under changing ecological pressures remains uncertain.

The expanding impact of climate change and environmental disruption represents another critical challenge. Alterations in rodent population dynamics, biodiversity, land use, and urban expansion are likely to continue reshaping hantavirus ecology across multiple continents. Increasing global mobility and travel‐associated clusters may facilitate the geographic dissemination of hantavirus infections beyond traditionally endemic regions, while climate change, biodiversity disruption, and expanding rodent‐human interfaces may increase future spillover events and complicate outbreak prediction. Predictive models integrating environmental surveillance, rodent ecology, and human epidemiology may therefore become increasingly important for outbreak forecasting and preparedness [2, 35, 37, 39, 40, 41, 43, 44, 66].

Substantial uncertainties also remain regarding long‐term outcomes following hantavirus infection. Persistent fatigue, renal impairment, cardiopulmonary dysfunction, and reduced quality of life have all been described among survivors, but the mechanisms underlying these chronic sequelae remain insufficiently characterised [78, 80]. Hantaviruses, including PUUV, are also capable of infecting B lymphocytes and inducing polyclonal B‐cell activation, and large register‐based cohort studies from Finland and Sweden have reported a two‐to three‐fold increased risk of lymphoid malignancies in the first one to 5 years after PUUV infection [92]; an increased incidence of other malignancies has similarly been reported after HTNV infection, although the biological basis of these associations requires further study. Beyond capillary leak, several additional mechanisms, including direct viral cytopathic effects on tubular epithelium, immune‐complex deposition, and complement activation, have also been proposed to contribute to hantavirus‐associated kidney dysfunction, alongside endothelial permeability changes. From a therapeutic perspective, the absence of validated targeted treatments continues to represent a major unmet clinical need. Future progress will likely depend on integrating advances in structural biology, immunology, and translational medicine to develop effective antivirals, monoclonal antibodies, and vaccine platforms capable of broad protection across hantavirus species [25, 26, 27, 28, 29, 30, 31, 32, 33, 34].

Finally, hantavirus disease increasingly illustrates the limitations of traditional siloed approaches to emerging infections. Effective preparedness will require integrated One Health frameworks capable of combining wildlife surveillance, climate science, molecular epidemiology, critical care preparedness, and coordinated international public health response. Integrated One Health surveillance systems that combine clinical, ecological, veterinary, and environmental data are likely to become increasingly important for early warning and preparedness strategies [2, 35, 37, 38, 45, 62, 63].

Hantavirus infection represents more than a rodent‐borne zoonosis. It illustrates how environmental change, viral evolution, and global interconnectedness can reshape the epidemiology of emerging pathogens. Future preparedness will therefore depend not only on biomedical innovation, but also on integrated One Health approaches capable of linking ecological surveillance, clinical medicine, molecular epidemiology, and coordinated public health response.

## Author Contributions

**Francesco De Maria:** conceptualisation, investigation, writing – original draft preparation, writing – review and editing. **Francesco Branda:** conceptualisation, investigation, writing – original draft preparation, writing – review and editing. **Giancarlo Ceccarelli:** investigation, writing – original draft preparation, writing – review and editing. **Fabio Scarpa:** investigation, writing – original draft preparation, writing – review and editing. **Massimo Ciccozzi:** conceptualisation, validation, supervision, writing – original draft preparation, writing – review and editing. **Alessandro Russo:** validation, supervision, writing – original draft preparation, writing – review and editing. All authors have read and agreed to the published version of the manuscript.

## Funding

The authors have nothing to report.

## Disclosure

All the authors contributed to the final article revision and approved the submitted version.

## Ethics Statement

This study is a narrative review based exclusively on previously published scientific literature. No new experiments involving human participants or animals were conducted, and no primary data were collected. All information analysed and discussed in this manuscript derives from peer‐reviewed studies that were performed in accordance with the ethical standards and regulatory requirements applicable at the time of their original publication.

## Conflicts of Interest

The authors declare no conflicts of interest.

## Supporting information


**Table S1:** Confirmed hantavirus disease cases by region, country, and virus over the past decade.

## Data Availability

Data sharing not applicable to this article as no datasets were generated or analysed during the current study.
