# Supplementary material for: The Changing Landscape of Hantavirus Infections: A Narrative Review of Epidemiology, Pathogenesis, and Countermeasures
Source: Rev Med Virol. 2026 Jul 31;36(5):e70191. doi: 10.1002/rmv.70191 (PMC13427222; doi:10.1002/rmv.70191)
Supplement: Supplementary file 1 — Table S1: Confirmed hantavirus disease cases by region, country, and virus over the past decade. [file RMV-36-e70191-s001.docx]

**The changing landscape of hantavirus infections: A narrative review of epidemiology, pathogenesis, and countermeasures**

**Authors**: Francesco De Maria^1^, Francesco Branda^2,*^, Giancarlo Ceccarelli^3^, Fabio Scarpa^4^, Massimo Ciccozzi^2^, Alessandro Russo^1^

**Affiliations**

1. Infectious and Tropical Diseases Unit, Department of Medical and Surgical Sciences, "Magna Graecia" University of Catanzaro, Catanzaro, Italy;
2. Unit of Medical Statistics and Molecular Epidemiology, Università Campus Bio-Medico di Roma, Rome, Italy;
3. Department of Public Health and Infectious Diseases, University Hospital Policlinico Umberto I, Sapienza University of Rome, Rome, Italy;
4. Department of Biomedical Sciences, University of Sassari, Sassari, Italy

**Correspondence***: Francesco Branda (f.branda@unicampus.it)

**Table S1. Confirmed hantavirus disease cases by region, country, and virus over the past decade.** Data are derived from national surveillance systems and may vary according to reporting practices, case definitions, and diagnostic capacity. HFRS = hemorrhagic fever with renal syndrome; HCPS = hantavirus cardiopulmonary syndrome; HTNV = Hantaan virus; SEOV = Seoul virus; PUUV = Puumala virus; SNV = Sin Nombre virus; ANDV = Andes virus.

| **Region/Country** | **Primary virus(es)** | **Period** | **Confirmed cases** | **Source** |
| --- | --- | --- | --- | --- |
| China | HTNV, SEOV | 2014–2023 | 91,388 (total) | China CDC Weekly (https://weekly.chinacdc.cn/fileCCDCW/journal/article/ccdcw/2025/25/PDF/CCDCW240640.pdf) |
| Finland | PUUV | 2020 | 1,166 | THL / Finnish National Infectious Diseases Register (https://sampo.thl.fi/pivot/prod/en/ttr/cases/fact_ttr_cases) |
|  |  | 2021 | 1,428 | THL / Finnish National Infectious Diseases Register (https://sampo.thl.fi/pivot/prod/en/ttr/cases/fact_ttr_cases) |
|  |  | 2022 | 1,299 | THL / Finnish National Infectious Diseases Register (https://sampo.thl.fi/pivot/prod/en/ttr/cases/fact_ttr_cases) |
|  |  | 2023 | 812 | THL / Finnish National Infectious Diseases Register (https://sampo.thl.fi/pivot/prod/en/ttr/cases/fact_ttr_cases) |
|  |  | 2024 | 586 | THL / Finnish National Infectious Diseases Register (https://sampo.thl.fi/pivot/prod/en/ttr/cases/fact_ttr_cases) |
|  |  | 2025 | 1,014 | THL / Finnish National Infectious Diseases Register (https://sampo.thl.fi/pivot/prod/en/ttr/cases/fact_ttr_cases) |
|  |  | 2026 | 559 (partial) | THL / Finnish National Infectious Diseases Register (https://sampo.thl.fi/pivot/prod/en/ttr/cases/fact_ttr_cases) |
| Sweden | PUUV | Annual average | 100–450 (hospitalized) | Folkhälsomyndigheten (https://www.folkhalsomyndigheten.se/statistik-och-data/hitta-statistik-och-data/virala-hemorragiska-febrar-statistik) |
| Germany | PUUV | 2020 | 237 | ECDC Annual Epidemiological Report (https://www.ecdc.europa.eu/sites/default/files/documents/HANTA_AER_2023.pdf) |
|  |  | 2021 | 1,535 | ECDC Annual Epidemiological Report (https://www.ecdc.europa.eu/sites/default/files/documents/HANTA_AER_2023.pdf) |
|  |  | 2023 | 335 | ECDC Annual Epidemiological Report (https://www.ecdc.europa.eu/sites/default/files/documents/HANTA_AER_2023.pdf) |
|  |  | 2026 | ≥9 (Lower Saxony, partial) | RKI (via Kreiszeitung, 6 May 2026, https://www.kreiszeitung.de/lokales/niedersachsen/toedliches-virus-drei-menschen-auf-kreuzfahrtschiff-gestorben-faelle-auch-in-niedersachsen-nachgewiesen-94293868.html) |
| United States | SNV | 1993–2024 | 890 | CDC (https://www.cdc.gov/hantavirus/data-research/cases/) |
| Argentina | ANDV | 2020–2021 | 54 | Infobae (citing Ministry of Health, https://www.infobae.com/salud/2026/05/06/hantavirus-en-argentina-se-notificaron-42-casos-en-lo-que-va-del-ano/) |
|  |  | 2021–2022 | 43 | Infobae (citing Ministry of Health, https://www.infobae.com/salud/2026/05/06/hantavirus-en-argentina-se-notificaron-42-casos-en-lo-que-va-del-ano/) |
|  |  | 2022–2023 | 63 | Infobae (citing Ministry of Health, https://www.infobae.com/salud/2026/05/06/hantavirus-en-argentina-se-notificaron-42-casos-en-lo-que-va-del-ano/) |
|  |  | 2023–2024 | 75 | Infobae (citing Ministry of Health, https://www.infobae.com/salud/2026/05/06/hantavirus-en-argentina-se-notificaron-42-casos-en-lo-que-va-del-ano/) |
|  |  | 2024–2025 | 57 | Infobae (citing Ministry of Health, https://www.infobae.com/salud/2026/05/06/hantavirus-en-argentina-se-notificaron-42-casos-en-lo-que-va-del-ano/) |
|  |  | 2025–2026 | 106 (as of SE 19) | Ministry of Health – BEN SE 19 (https://www.argentina.gob.ar/noticias/actualizacion-del-boletin-epidemiologico-nacional-de-la-semana-ndeg-19) |
| Chile | ANDV | 2020–2024 (average) | 30–70 annual | Public epidemiological reports (https://www.swissinfo.ch/spa/chile-registra-39-casos-de-hantavirus-en-lo-que-va-de-2026-y-un-aumento-de-la-letalidad/91376634) |
|  |  | 2025 | 44 | ISP / MINSAL (https://ispch.cl/noticia/isp-refuerza-que-se-mantiene-el-alto-riesgo-de-infeccion-por-virus-hanta/) |
|  |  | 2026 (as of 7 May) | 39 | Public epidemiological reports (https://www.swissinfo.ch/spa/chile-registra-39-casos-de-hantavirus-en-lo-que-va-de-2026-y-un-aumento-de-la-letalidad/91376634) |
